# Inattention, academic underachievement, and depressive symptoms: uncovering environmental and genetic pathways from middle to late childhood

**DOI:** 10.3389/frcha.2023.1113938

**Published:** 2023-06-14

**Authors:** André Plamondon, George M. Tarabulsy, Ginette Dionne, Isabelle Ouellet-Morin, Frank Vitaro, Mara Brendgen, Michel Boivin

**Affiliations:** ^1^ Department of Educational Fundamentals and Practices, Université Laval, Québec, QC, Canada; ^2^ School of Psychology, Université Laval, Québec, QC, Canada; ^3^ School of Criminology, Université de Montréal, Montréal, QC, Canada; ^4^ School of Psychoeducation, Université de Montréal, Montréal, QC, Canada; ^5^ Department of Psychology, Université du Québec à Montréal, Montréal, QC, Canada

**Keywords:** attention deficit and hyperactivity disorder (ADHD), depression, inattention, academic achievement, genetically sensitive design

## Abstract

**Introduction:**

School underachievement has been shown to mediate the association between inattention and depressive symptoms in middle childhood. However, is it not clear whether these sequential associations are underpinned by genetic and environmental pathways, and the extent to which associated disruptive behaviors, such as hyperactivity/impulsivity, and peer relation difficulties partly account for these associations.

**Methods:**

The present study used a longitudinal study of twins assessed from Kindergarten to Grade 6 to address these questions using multivariate biometric modeling.

**Results:**

The hypothesized genetically informed (twin) model revealed that over and above disruptive behaviors and relational difficulties, there was evidence for (1) shared genetic factors partly accounting for these associations, and for (2) putative phenotype-to-phenotype associations sequentially linking inattention, school achievement, and depressive symptoms.

**Discussion:**

Confirmation of the expected sequence of phenotype-to-phenotype associations (i.e., in addition to shared genetic factors) suggests an environmental pathway linking these phenotypes. The discussion focuses on the relevance and significance of these pathways for understanding the development of school and mental health problems, as well as for the identification of children at risk and early preventive interventions.

## 1. Introduction

Attention deficit and hyperactivity disorder (ADHD), which is manifested by inattention and hyperactivity/impulsivity symptoms, has been linked to depressive symptoms in childhood (1). However, it is not clear why this specific association arises. The dual-failure model postulates that early externalizing behaviors, including symptoms of ADHD, lead to adjustment problems within the school context, which, in turn, may contribute to the development of internalizing difficulties, such as depressive symptoms (2–6). Thus, middle childhood appears a crucial period for the emergence of an association between externalizing and internalizing behaviors, and any sound empirical evaluation of this model must start at school entry and extend longitudinally to document how these associations unfold.

The dual-failure model posits that the association between externalizing and internalizing behaviors results from failures in two spheres of functioning: peer relationships and academic achievement. Previous findings have mainly focused on the peer relationship pathway and found that it was mostly accounted for by disruptive behaviors, such as aggression (7, 8). The second putative pathway, involving academic achievement, may be more relevant in accounting for the association between ADHD and depressive symptoms. Indeed, previous findings suggest that the association between externalizing difficulties and academic achievement may be due in large part to symptoms of ADHD, mostly inattention (9, 10). Furthermore, inattention, but not conduct disorder, has been associated with depressive symptoms in childhood (11, 12), thus suggesting that this association is independent from other forms of externalizing behaviors. Therefore, the dual-failure model may represent two distinct, but correlated pathways leading to internalizing difficulties: one involving disruptive behaviors and peer difficulties, and a second involving inattention and academic underachievement.

There is direct evidence supporting a development pathway linking inattention, academic achievement, and depressive symptoms that is independent from co-occurring disruptive behaviors. In a sample of children followed from Grade 1 to Grade 3 (11), inattention was shown to predict academic achievement, which then predicted depressive symptoms. Moreover, conduct problems, a class of disruptive behaviors, were not associated with academic achievement nor depressive symptoms. Given the strong associations, temporal precedence, and control for confounders, these results were interpreted as pointing to a causal role of inattention in the genesis of academic difficulties and depressive problems (13). However, hyperactive/impulsivity symptoms, a central dimension of ADHD, were not assessed in this study. While there is solid evidence that symptoms of ADHD are associated with academic achievement through inattention rather than hyperactivity/impulsivity (14, 15), the symptoms accounting for the association with depressive symptoms are less clear. The primary aim of this paper was to document whether inattention leads to academic underachievement, and then to depressive symptoms in childhood, while accounting for the contribution of hyperactivity/impulsivity.

### 1.1. The roles of genetic and environmental factors as underlying factors

To the extent that there is a consequential phenotypic pathway linking inattention, academic underachievement, and depressive symptoms, it is not clear whether this developmental sequence is environmentally or genetically mediated. Indeed, genetically informed studies raise the possibility that a combination of shared etiological factors could account for these associations. Evidence of shared genetic factors could reflect two possibilities. First, correlated genetic factors could be involved through a third variable, such as some form of biological endophenotype that is responsible for the phenotypic associations. Second, there could be an actual developmental sequence whereby genetic factors underlying inattention extend to academic underachievement, and thus the observed associations. For instance, a child could experience academic difficulties due to its genetic vulnerability for inattention. On the other hand, a lack of evidence for shared etiological factors in the presence of significant phenotypic associations would indicate a phenotype-to-phenotype linkage consistent with a causal association. Clarifying whether the observed associations are accounted for by shared etiological factors could help identify potential intervention targets. If shared etiological factors are found to account for the trivariate associations, then investigating further putative biosocial mechanisms underlying these associations will help orient preventive intervention strategies. If direct phenotype-to-phenotype associations are found, then interventions could potentially target each phenotype directly to prevent its cascading effects.

There is indeed evidence that shared etiological factors are implicated in the associations between ADHD symptoms, academic achievement, and depressive symptoms. First, the association between inattention or symptoms of ADHD and depressive symptoms in children and adolescents was found to be mostly accounted for by genetic overlap, although there was also evidence of overlap in shared and non-shared environmental factors (16–18). Second, most previous studies found that the association between inattention, or more generally symptoms of ADHD and academic achievement in childhood is mainly, if not entirely, accounted by shared genetic factors (10, 19, 20). One study, however, found evidence of a phenotype-to-phenotype association between symptoms of ADHD and academic achievement, even after accounting for shared etiological factors (21).

The final bivariate association of the hypothesized academic failure pathway, between academic underachievement and depressive symptoms, has never been examined in a genetically informative sample. Although children with a reading disability were found to be at increased risk for internalizing behaviors compared to a community control group, their siblings with a normal reading ability were not (22). The lack of association between a child's reading disability and his or her sibling's internalizing behaviors indicates that the association between reading disability and internalizing problems was not accounted for by shared familial factors (genetic or environmental). Rather, reading difficulties appeared as an individual-level risk factor for internalizing behaviors. This suggests that the association between academic achievement and depressive symptoms is child-specific and perhaps environmental.

While genetically informed designs are useful to document the roles of unobserved genetic and environmental factors in the observed associations, they can still be contaminated by confounders. Taking into account potential confounders is thus an important step in specifically testing the sequence of associations between inattention, academic underachievement, and depressive symptoms as a distinct environmental pathway, especially within the dual-pathway model. Here, two risk factors were considered as controls in the model. The first risk factor to be controlled for was disruptive behaviors, that is externalizing behaviors (aggression, conduct problems, oppositionality) excluding ADHD-related symptoms. Disruptive behaviors are strongly associated with ADHD and depressive symptoms (23, 24), as well as with academic underachievement (25). The other risk factor that was controlled was relational difficulties, which encompassed both peer- and teacher-related difficulties to provide a comprehensive coverage of relational problems. Indeed, relational difficulties may negatively contribute to both academic achievement and depressive problems (13). Consistent with the dual-failure model, poor peer relationships have been associated with academic underachievement (26, 27) and internalizing difficulties (6). Teacher-child relationships have been negatively associated with academic achievement (27), as a protective factor against depressive problems for children with pre-existing difficulties (28), as well as rated as more stressful when dealing with children with ADHD (29). All of this points to their relevance in the context of symptoms of ADHD and its possible consequences.

### 1.2. The present study

The objectives of this study were three-fold. First, we wanted to test across childhood (Kindergarten to Grade 6) whether inattention symptoms were longitudinally associated with depressive symptoms via academic achievement, after accounting for hyperactivity/impulsivity. Because developmental models have highlighted that the cascade between externalizing and internalizing occurs during elementary school, we studied a sample of children. Second, we wanted to test whether this putative developmental pathway was accounted for by shared genetic or environmental factors. Third, we wanted to investigate the contribution of three potential risk factors (victimization, teacher-child relationship, and disruptive behaviors) to the hypothesized developmental model. We hypothesized that inattention would be associated with depressive symptoms via academic achievement, and after accounting for hyperactivity/impulsivity. Given previous results supporting the contribution of genetic factors to the associations between symptoms of ADHD and academic achievement, as well as between ADHD and mood problems, we hypothesized that their associations would be mostly accounted for by shared genetic factors. Finally, we hypothesized that the associations between inattention, academic achievement, and depressive symptoms would not be substantially changed when accounting for disruptive behaviors, as well as peer and teacher-child relationship difficulties.

## 2. Materials and methods

### 2.1. Sample

The current sample was part of the Quebec Newborn Twin Study (QNTS), an ongoing population-based longitudinal study of 630 twin pairs born in the greater Montreal area between November 1995 and July 1998. Children were assessed on a variety of child-, family-, and school-related characteristics from 6=months onward (30). Approval from the ethics committee of the participating universities and informed parental consent was obtained before each wave of data collection. The QNTS families were similar on sociodemographic data to families from a birth cohort representative of the large urban centers of Québec (30). Zygosity was ascertained by two independent evaluators using the Zygosity Questionnaire for Young Twins when the twins were 18 months old (31, 32). In cases where questionnaire-based zygosity was inconclusive, DNA analyses were performed. The current sample was composed of 219 monozygotic (MZ) twin pairs and 315 dizygotic (DZ) twin pairs who were evaluated in Kindergarten (K; mean age = 6.04 years), Grade 1 (G1; mean age = 7.08 years), Grade 3 (G3; mean age = 9.10 years), Grade 4 (G4; mean age = 10.00 years), and Grade 6 (G6; mean age = 12.09 years).

### 2.2. Measures

#### 2.2.1. Inattention and hyperactivity/impulsivity

Teachers rated children using items from the Social Behavior Questionnaire (33), a reliable and valid behavior problem inventory (34–36), which was partly based on the Children's Behavior Questionnaire (37) and the Preschool Behavior Questionnaire (38). Teachers rated the frequency of each symptom on a 3-point Likert scale: 0 = never; 1 = sometimes; and 2 = often. Inattention symptoms were assessed by using three items (*α* = 0.89 in Kindergarten, 0.90 in Grade 1): “…was easily distracted, had trouble sticking to an activity?”; “…was unable to concentrate, could not pay attention for long?”; and “…was inattentive?” Hyperactivity/impulsivity symptoms were assessed through five items (*α* = 0.90 in Kindergarten, 0.89 in Grade 1), such as “… couldn’t stop fidgeting?” and “… was impulsive, acted without thinking?” Both inattention and hyperactivity/impulsivity scores were substantially correlated between Kindergarten and Grade 1 (*r* = 0.52 and 0.60, respectively) and were therefore aggregated into mean scores.

#### 2.2.2. Academic achievement

In the spring of Grades 3 and 4, teachers were asked to evaluate a child's school achievement in mathematics, reading, writing, and overall academic achievement relative to his or her classmates, using five items (Grade 3: *α* = 0.94) and seven items (Grade 4: *α* = 0.95). The teachers rated how the child fared compared to its peers, from 1, clearly under average, to 5, clearly above average. This rating method has been shown to be associated with actual achievement (39, 40). Given the high correlation between the two grade-specific scales (*r* = 0.62), they were aggregated. Teacher ratings of school achievement are generally reliable and valid, as indicated by their substantial correlations with other measures of academic achievement scores, including standardized measures of performance. A meta-analysis estimated that teacher's assessments of student's school achievement is highly related to their actual test performance (average *r *= 0.63) (41).

#### 2.2.3. Depressive symptoms

Children were asked to report on their depressive symptoms in Grade 6. The scale was based on seven items from the Children's Depression Inventory (42), each rated on a 3-point scale, which were averaged to create a summary score. Reliability was moderate (*α* = 0.68). The Children's Depression Inventory scale is often used to measure childhood depressive symptomatology and is sensitive to developmental and gender differences in depressive symptoms from childhood onward (43). It was also shown to be valid as a screening measure for depression (44).

#### 2.2.4. Control variables

To control for the contribution of various socio-emotional variables, we used the following scales, all reported when children were in Grade 1. Victimization was reported by teachers using three items: “was called names by other children”; “was hit or pushed by other children”; and “was made fun of by other children” (*α* = 0.68). This scale was shown to be associated with parent-rated trajectories of preschool victimization (7), suggesting that it adequately represents a child's experience of victimization. Teacher-child relationship quality was evaluated using the Conflict and Closeness subscales of the Teacher-Child Relationship Scale (STRS) (45). We used a total of four items that assess the closeness, warmth, and conflict of their relationship (*α* = 0.73). This scale was shown to predict changes in academic achievement at the beginning of the school period (27). Finally, disruptive behaviors were reported by teachers in Grade 1. This scale comprised 11 items from the Social Behavior Questionnaire (30) which measured oppositional behaviors and conduct problems, such as cheating, lying, and physical aggression (*α* = 0.84).

### 2.3. Analyses

All models were fitted to standardized sex-regressed variables if there were gender differences, as assessing gender differences was not the object of the current study. The structural equation modeling program M*Plus* 8.9 (46) was was used for all analyses. The use of full-information maximum likelihood allowed the incorporation of subjects with missing data under the missing at random assumption. To make this assumption more plausible, we incorporated auxiliary variables in the models (47). Since the data were slightly non-normal, we used an estimation method robust to non-normality, the maximum-likelihood estimator with robust standard errors, as was done elsewhere with twin data (18). Models were compared using the likelihood ratio test, which compares the goodness-of-fit, as measured by the Yuan–Bentler (Y–B) χ^2^, between a baseline model and a more restrictive nested model. The comparison between models was done using the change in the Y–B χ^2^ test. The degrees of freedom for this test are equal to the difference between the number of parameters in the baseline model and the number of parameters in the more restrictive nested model. Relative fit indices were also used to assess model fit. We used the Comparative Fit Index (CFI), Tucker–Lewis Index (TLI), and Root-Mean Square Error of Approximation (RMSEA). For the CFI and TLI, a value closer to 1 indicates a better fit. Cut-off values of 0.950 have been suggested (48). For the RMSEA, a value <0.050 indicates a close approximate fit (48).

The twin method relies on the difference in genetic relatedness between pairs of MZ twins, who share 100% of their genes, and DZ twins, who share, on average, 50% of their genes. The differing degree of genetic relatedness allows us to estimate the relative contribution of genetic and environmental factors to the total phenotypic variance. The total phenotypic variance is typically decomposed into additive genetic factor (*A*), shared environmental factors (*C*), and non-shared environmental factors (*E*). Additive genetic factors reflect the sum of the effect of two or more gene loci. Shared environmental factors reflect environmental factors that make twins similar, regardless of zygosity. Finally, non-shared environmental factors are environmental factors unique to each child that make twins more dissimilar. Non-shared environmental factors also contain measurement error as they contribute to make twins dissimilar, regardless of zygosity.

For our first objective, we tested the hypothesized mediation model using a phenotypic analysis controlling for hyperactivity/impulsivity in Kindergarten/Grade 1 (i.e., ignoring the genetically sensitive design of the sample). We used a sandwich estimator to account for the nesting of children within families (46). Models with the robust standard errors were used to derive model fit indices, but all parameter estimates as well as their 95% confidence intervals (95% CIs) were derived by running the models with 5,000 bootstrap samples (49). An association was considered significant if its 95% CI did not include zero.

**Figure 1:**
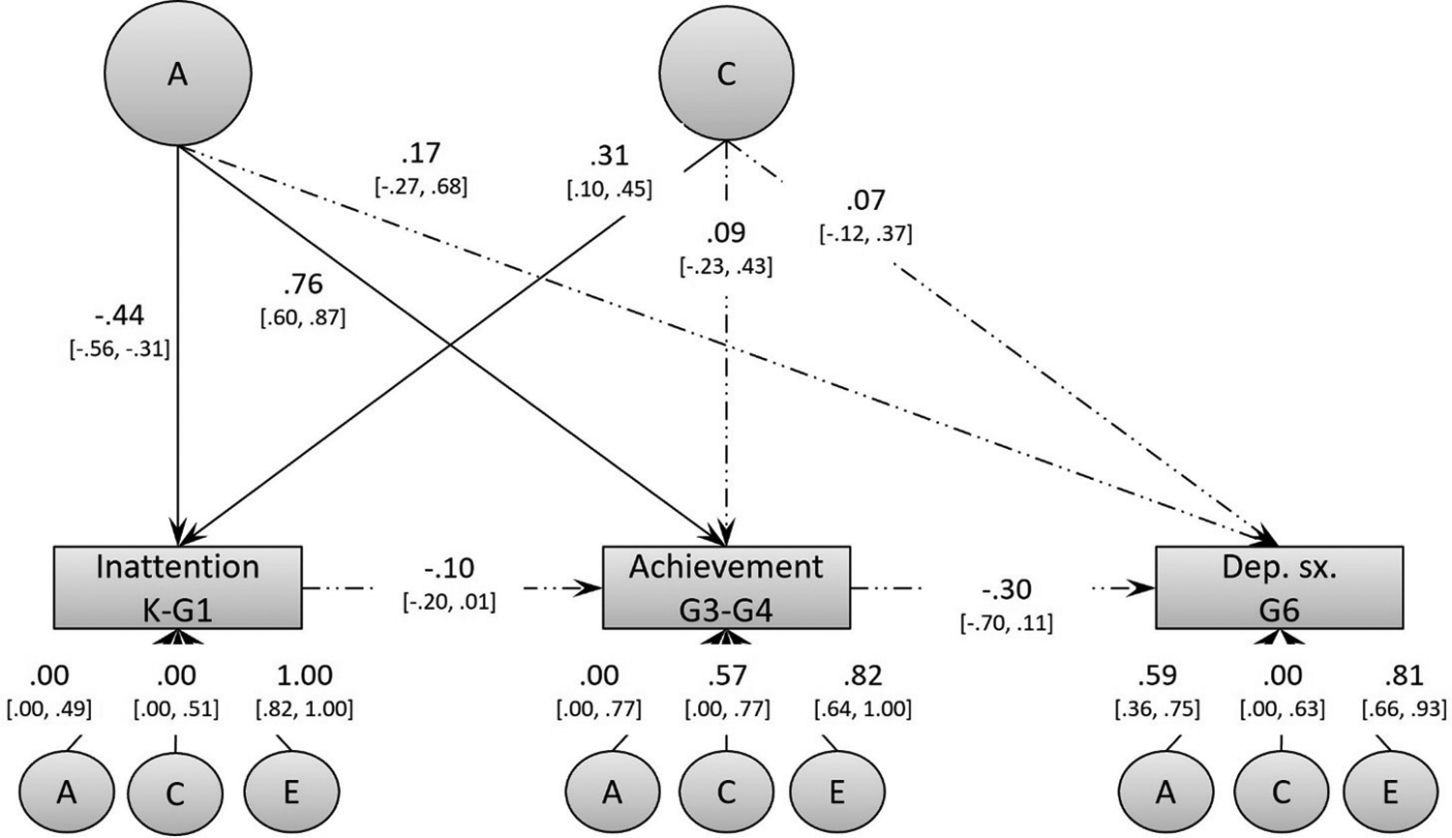
Baseline biometric model (Model 1) of the associations between inattention, academic achievement, and depressive symptoms. This model controls for hyperactivity/impulsivity, but these effects are not shown in the figure to reduce clutter. Dep. sx. = depressive symptoms.

The second objective was to identify possible phenotype-to-phenotype associations, while controlling for shared genetic and environmental factors. This model, depicted in Figure 1, uses multivariate biometric modeling, and is an extension of a model originally developed to decompose the association between two variables (50). Variances and covariances were decomposed into additive genetic effects, shared environmental effects, and non-shared environmental effects (labeled *A*, *C*, and *E*, respectively). Here, we used an independent pathway model given the previous evidence of genetic and environmental factors accounting for the associations between the variables under study. Shared etiological factors between inattention, academic achievement, and depressive problems are depicted in Figure 1. As in the original model (50), we did not include a common non-shared environmental factor. Residual variance for each phenotype was further decomposed into genetic and environmental sources of variance. Note that all models controlled for hyperactivity/impulsivity. Phenotype-to-phenotype associations were modeled using partial regression coefficients from inattention to academic achievement, and then from academic achievement to depressive symptoms. If shared etiological factors fully accounted for the associations between all variables, these partial regression coefficients should be non-significant. For a more technical description of this model, see Kohler et al. (50).

## 3. Results

Bivariate associations, as well as means and SD, are presented in Table 1. As hypothesized, inattention in Kindergarten/Grade 1 was more strongly correlated with academic achievement in Grade 3/4 than hyperactivity/impulsivity in Kindergarten/Grade 1. Conversely, hyperactivity/impulsivity in Kindergarten/Grade 1 was more strongly associated with victimization and disruptive behaviors at the same age. Teacher-child relationship quality in Grade 1 was slightly more associated with inattention than hyperactivity/impulsivity, although the difference was small in magnitude. In addition, academic achievement in Grade 3/4 was the strongest correlate of self-reported depressive symptoms in Grade 6. Twin correlations are also included in Table 2.

**Table 1:** Descriptive statistics.

	1.	2.	3.	4.	5.	6.	7.	M	SD
1. Inattention								0.82	0.62
2. Academic achievement	−0.51*							3.05	1.02
3. Depressive symptoms	0.13*	−.017*						1.25	0.28
4. Hyperactivity-impulsivity	0.65*	−0.25*	0.12*					0.52	0.53
5. Victimization	0.36*	−0.24*	−0.24*	0.43*				0.28	0.37
6. Teacher-child relationship	−0.29*	0.19*	−0.01	−0.28*	−0.26*			4.24	0.62
7. Disruptive behaviors	0.42*	−0.22*	0.07	0.66*	0.54*	−0.45*		0.31	0.32

* *p* < 0.05.

**Table 2:** Twin correlations.

Measure	MZ		DZ	
	Estimate	95% CI	Estimate	95% CI
**Cross-twin correlations**				
Inattention	0.60*	0.49 to 0.69	0.37*	0.26 to 0.47
Hyp./Imp.	0.68*	0.56 to 0.77	0.32*	0.20 to 0.42
Achievement	0.78*	0.70 to 0.85	0.46*	0.35 to 0.56
Dep. Sx.	0.34*	0.18 to 0.50	0.18*	0.03 to 0.36
**Cross-trait cross-twin correlations**				
Inattention—Hyp./Imp.	0.48*	0.37 to 0.56	0.25*	0.16 to 0.34
Inattention—Achievement	−0.42*	−0.51 to −0.31	−0.29*	−0.38 to −0.19
Inattention—Dep. Sx.	0.06	−0.06 to 0.18	0.10	0.00 to 0.20
Hyp./Imp.—Achievement	−0.16*	−0.26 to −0.04	−0.20*	−0.29 to −0.11
Hyp./Imp.—Dep. Sx.	0.08	−0.04 to 0.21	0.06	−0.04 to 0.16
Achievement—Dep. Sx.	−0.02	−0.15 to 0.13	−0.17*	−0.25 to −0.08

Hyp./Imp. = hyperactivity/impulsivity; Dep. Sx. = depressive symptoms; MZ, monozygotic; DZ, dizygotic.

* *p* < .05.

**Figure 2:**
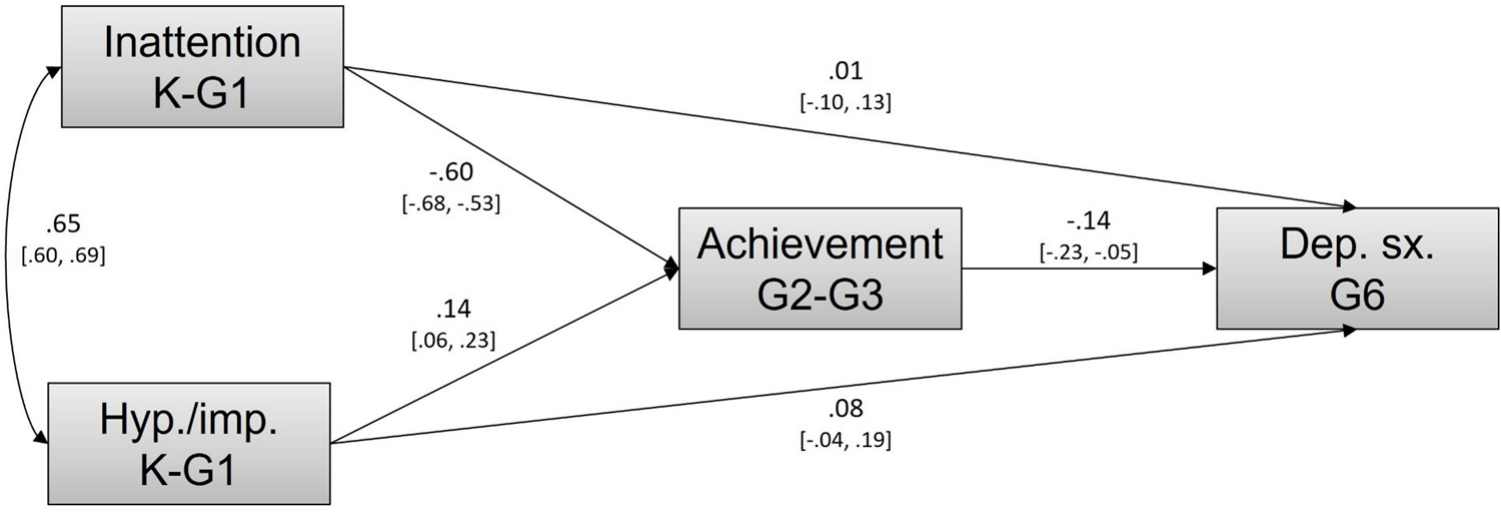
Phenotypic mediation model linking inattention, academic achievement, and depressive symptoms while controlling for hyperactivity/impulsivity.

Regarding our first objective, we tested the hypothesized mediation model using a phenotypic analysis controlling for hyperactivity/impulsivity in Kindergarten/Grade 1. This model, depicted in Figure 2, was fully saturated and therefore fitted the data perfectly, Y–B χ^2^ (0) = 0, *p* > 0.05, CFI = 1.00, TLI = 1.00, RMSEA = 0.000. The indirect association between Kindergarten/Grade 1 inattention and Grade 6 depressive symptoms via lower Grade 3/4 academic achievement was significant (β = 0.084, 95% CI 0.028–0.142). Results also showed that the direct association between inattention and depressive symptoms was virtually null, suggesting that their apparent association was entirely accounted for by lower academic achievement. This analysis also revealed that the association between hyperactivity/impulsivity and academic achievement became *positive* once the contribution of inattention was taken into account, thus suggesting some suppressor effect in the case of hyperactivity/impulsivity. Accordingly, further analyses only included hyperactivity/impulsivity as a covariate.

For our second objective, we tested the phenotype-to-phenotype associations between inattention, academic achievement, and depressive symptoms under conditions of genetic-environmental control using biometric modeling. The full baseline model described earlier can be seen in Figure 1. This model fitted the data well (see Model 1 in Table 3). Based on this model, we derived our final model by dropping the following non-significant paths: the shared common environmental factor; the shared genetic factor underlying depressive symptoms; the genetic components for inattention and for academic achievement; and the shared environmental variance for depressive symptoms. Model 2 did not significantly differ from Model 1 (ΔY–B χ^2^ (7) = 3.22, *p* > 0.05), and was thus retained as it was deemed more parsimonious.

**Table 3:** Fit indices of the different biometrical models.

	Y–B *χ*^2^	Scaling	*df*	CFI	TLI	RMSEA
Model 1: baseline model	86.159*	1.16	61	0.973	0.976	0.039
Model 2: final model	92.923*	1.10	68	0.973	0.979	0.037
Model 3: model 2 + covariates	123.846*	1.09	95	0.970	0.976	0.034

Y–B χ^2^ = Yuan–Bentler χ^2^; CFI, Comparative Fit Index; TLI, Tucker–Lewis Index; RMSEA, Root-Mean Square Error of Approximation.

* *p* < .05.

All models control for hyperactivity/impulsivity.

**Figure 3:**
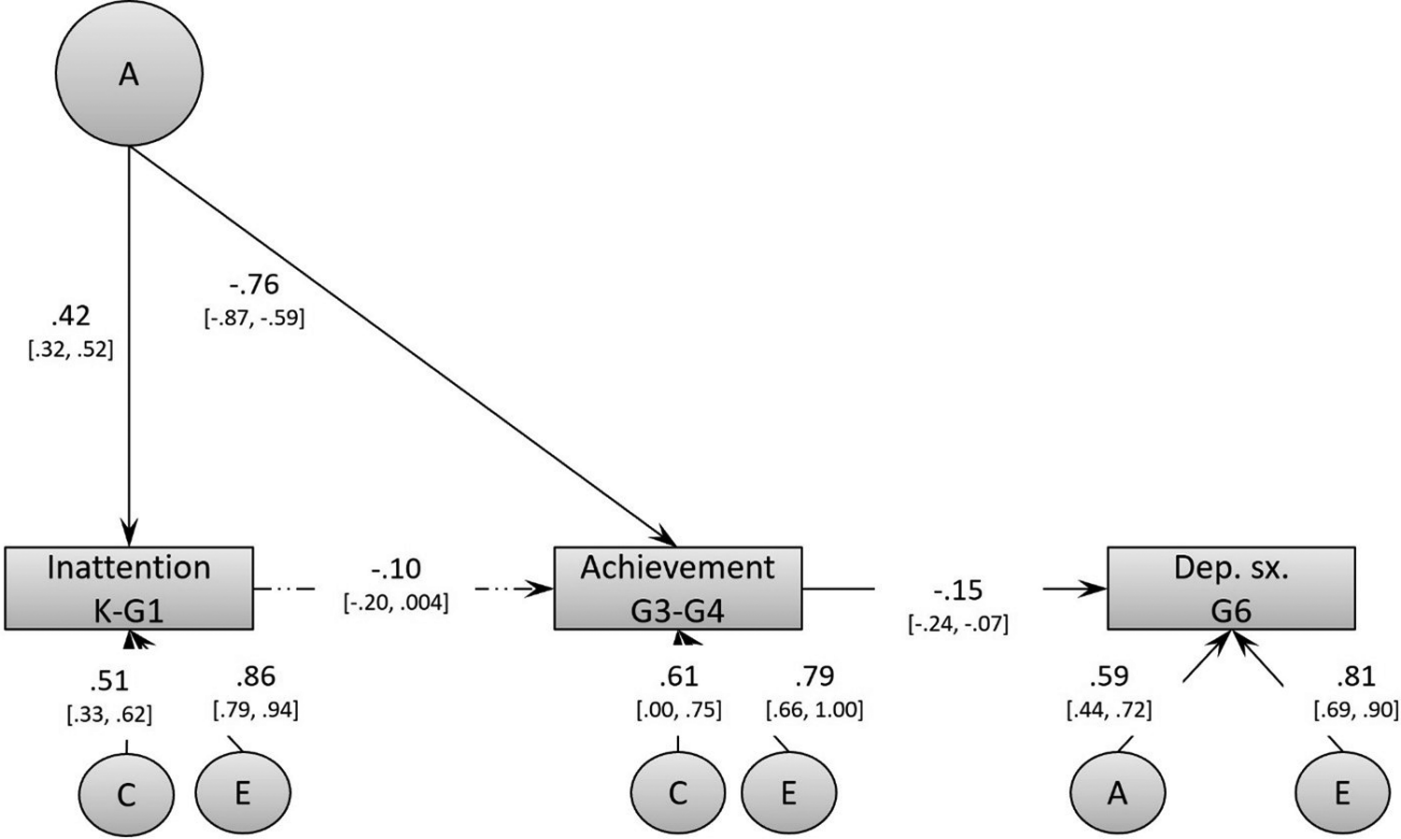
Final biometric model (Model 2) of the associations between inattention, academic achievement, and depressive symptoms. This model controls for hyperactivity/impulsivity, but these effects are not shown in the figure to reduce clutter.

The final, best-fitting model is depicted in Figure 3. The model reveals common genetic variance between early inattention (Kindergarten/Grade 1) and later academic achievement (Grade 3/4). Using path-tracing rules, we found that genetic factors accounted for a phenotypic association of *r* = −0.34 (i.e., 0.42 × −0.76) between these two constructs. However, depressive symptoms in Grade 6 were not associated with these genetic factors. Thus, the association between Grade 3/4 academic achievement and Grade 6 depressive symptoms could not be accounted for by these shared etiological factors. It is notable that the shared genetic variance entirely accounted for the genetics underlying inattention and academic achievement. Following path-tracing rules, the proportion of variance accounted for by genetic and non-shared environmental variance can be derived by squaring their respective path (although they do not equal exactly to 100% due to rounding). Thus, after accounting for the common genetic variance and the phenotype-to-phenotype association, shared and non-shared environmental factors accounted for approximately 26% and 74%, respectively, of the residual variance in inattention, as well as for approximately 37% and 63%, respectively, of the residual variance of academic achievement. Similarly, residual variance in depressive symptoms in Grade 6 were also accounted for by both genetic (approximately 35%) and non-shared environmental variance (approximately 65%).

### 3.1. Phenotype-to-phenotype associations

Most importantly, over and above shared genetic factors, the phenotype-to-phenotype association between Kindergarten/Grade 1 inattention and Grade 3/4 academic achievement fell short of significance (β = −0.10, 95% CI −0.20 to 0.004), whereas that between academic achievement and depressive symptoms was significant but modest (β = −0.15, 95% CI −0.24 to −0.07). After controlling for shared etiological factors, the indirect association between inattention and depressive symptoms was still significant, but much smaller than in the phenotypic analysis (β = 0.015, 95% CI 0.001–0.040).

### 3.2. Robustness to covariates

Covariates were then added to investigate their possible contribution to the hypothesized developmental model. In Model 3, we controlled for victimization, teacher-child relationship quality, and finally disruptive behaviors. The fit indices of this model, reported in Table 3, indicate that it fits the data well.

The results showed virtually no change in parameter estimates after controlling for all three covariates. The phenotypic association between inattention and academic achievement accounted for by genetic factors was almost identical (*r* = −0.31 compared to *r* = −0.34 before). The phenotype-to-phenotype associations were almost unchanged between inattention and achievement (β = −0.09, 95% CI −0.19 to 0.01), as well as between achievement and depressive symptoms (β = −0.15, 95% CI −0.23 to −0.06). The developmental cascade resulting from both phenotype-to-phenotype associations was similar in size, but fell short of significance (β = 0.013, 95% CI 0.000–0.037).

## 4. Discussion

The present study had three main objectives. First, we wanted to test across childhood (Kindergarten to Grade 6) whether inattention symptoms were longitudinally associated with depressive symptoms via academic achievement, after accounting for hyperactivity/impulsivity. Second, we wanted to test whether this putative developmental pathway was accounted for by shared genetic and environmental factors, and whether there was evidence of phenotype-to-phenotype associations over and above these common etiological underpinning. Third, we wanted to assess the contribution of potential risk factors pertaining to relational difficulties (peer- and teacher-related) and disruptive behaviors to the expected developmental associations. Overall, and as expected, the hypothesized model revealed that over and above disruptive behaviors and relational difficulties, (1) there was evidence for shared genetic factors partly accounting for these associations, as well as (2) mixed evidence for the putative phenotype-to-phenotype associations sequentially linking inattention, school achievement, and depressive symptoms. Our results suggest that a mixture of genetic and environmental pathways link these phenotypes.

Regarding the first objective, our phenotypic analyses showed that inattention was associated with depressive symptoms via academic underachievement, even after accounting for hyperactivity/impulsivity. These findings are consistent with a previous study that also found support for this indirect pathway (11) but extend these previous results by showing that it is not accounted for by hyperactivity/impulsivity. The finding of a positive association between hyperactivity/impulsivity and achievement after accounting for inattention is intriguing as it suggests a suppressor effect (51). Such a variation in associations is not new, as the evidence for a link between lower academic achievement and hyperactivity/impulsivity is typically mixed across studies, but robust for inattention (52). This underlies the need for disaggregating inattention and hyperactive/impulsive symptoms when studying their developmental correlates.

With regard to the second objective, we found mixed evidence for phenotype-to-phenotype associations between inattention, academic achievement, and depressive problems above and beyond shared etiological factors. Although the indirect association was significant when accounting for shared etiological factors, this indirect association was quite reduced when accounting for the contribution of shared etiological factors (β = 0.015 vs. β = 0.084). This indicates that most of this mediating pathway was accounted for by shared genetic factors between inattention and lower academic achievement. The development of effective preventive intervention strategies could be guided by further uncovering the mechanisms accounting for the genetic factors shared between inattention and academic underachievement.

The mixture of genetic and environmental factors shared between inattention and academic achievement was expected and consistent with the dual pathways accounting for the association between ADHD and academic achievement (53). The first pathway, involving cognitive processes, may reflect the genetic association, and is consistent with previous findings showing that processing speed accounted for the genetic association between inattention and reading underachievement (54). Whether this cognitive skill also accounts for the association between inattention and academic underachievement remains to be confirmed. The second pathway, involving behaviors reflecting lower engagement toward schoolwork, may here be found in the phenotype-to-phenotype association between inattention and academic achievement. It suggests that, regardless of their genetic risk, inattentive children may miss learning opportunities. Consistent with this hypothesis, the association between school engagement and grades has been shown to be mostly accounted for by non-shared environmental factors (55). The small magnitude of this phenotype-to-phenotype association is to be expected given the consistently small associations among non-shared environmental factors (56). Whether this dual-pathway model accurately accounts for the genetic and environmental associations between inattention and academic underachievement remains to be evaluated.

One notable finding pertains to the significant phenotype-to-phenotype association between academic achievement and depressive symptoms. This phenotypic association has been supported in previous studies (5, 6, 57), but this is the first study to consider the possible shared etiological factors underlying this association. Our results suggest an active environmental role of lower academic achievement in the genesis of depressive problems.

Contrary to our expectations, we did not find evidence of shared genetic factors between early inattention and later depressive symptoms. There is indeed evidence that the genetic factors underlying depressive symptoms are shared with hyperactivity/impulsivity and comorbid inattention (18). We note, however, that our measures of inattention and depressive symptoms were reported by different informants, as well as over a few years. Given that concurrent associations between children's reports of internalizing difficulties and teacher reports are small to moderate (*r* = 0.20) (58), it is not surprising that we find a small indirect association. We do not interpret our findings as evidence of a lack of shared genetic underpinnings between inattention and depressive symptoms, but rather as being a conservative estimate of the putative environmental pathway linking inattention and depressive symptoms in childhood. If such an environmental mechanism exists, it may become stronger in late childhood, when self-appraisals of academic competence become more stable and more strongly related to teachers’ appraisals (59). It will be important to investigate this developmental pathway further over different periods of time and using different informants.

Perhaps more surprising was the lack of change in the results when accounting for early relational problems and disruptive behaviors. We expected inattention to be uniquely associated with academic achievement longitudinally, but we did not expect this association to be so robust to the addition of covariates. Rather, these difficulties were expected to play a significant role in the model given their documented shared genetic and environmental factors with academic achievement and depressive problems (60–63). Although the phenotype-to-phenotype mediation became marginally significant, there was little change in its effect size after adding covariates. Further studies are needed to further test whether the dual-failure model represents two distinct but correlated developmental pathways stemming from inattention and disruptive behaviors.

This study is not without limitations. One limitation is the reliance on teacher ratings for most measures as they provide an external, indirect account of peer processes and child experiences. However, from a clinical and research perspective, we contend that this may also be a strength, as raters varied every year and may facilitate generalizations to clinical assessments, which often rely on teacher reports. A second limitation concerns the developmental period under study. While we used a longitudinal design that covers middle and late childhood, we did not cover adolescence, a period during which the prevalence of depressive disorder increases (64). It will be important to investigate whether our results also apply during adolescence. By extension, it would be interesting to investigate whether the roles of different components of externalizing behaviors in the dual-pathway model change with age. Finally, our study did not include any molecular genetics, neuroimaging, or peripheral biomarker for in-depth phenotyping of symptoms of ADHD.

## 5. Conclusion

Our results suggest that inattention directly predicts academic underachievement, with the latter posing an environmental risk for increased depressive symptoms. This sequential pattern of associations stood after controlling for shared etiological and measured risk factors. The indirect association between inattention and depressive symptoms was partly accounted for by shared genetic factors but was still significant albeit much smaller. These patterns of associations were largely independent of concurrent relational and behavioral difficulties, raising the possibility that inattention and academic achievement represent a distinct pathway leading to depressive problems. Further research should evaluate the separate contribution of inattention and disruptive behaviors within the context of the dual-failure model.

## Data Availability

The data analyzed in this study are subject to the following licenses/restrictions: data cannot be shared publicly because they are proprietary but may be obtained by filling a request to access from the Research Unit on Children's Psychosocial Maladjustment Website. Requests to access these datasets should be directed to http://www.gripinfo.ca/grip/public/www/etudes/en/dadprocedures.asp.
